# Choroidal nevus and polypoidal vasculopathy: case series

**DOI:** 10.1186/s40942-020-00223-2

**Published:** 2020-06-02

**Authors:** Karlos Ítalo S. Viana, Pedro F. Dalgalarrondo, Zelia Correa, Rodrigo Jorge

**Affiliations:** 1 Department of Ophthalmology, Otorhinolaryngology and Head and Neck Surgery, School of Medicine of Ribeirão Preto, University of São Paulo, Av. Bandeirantes 3900, Ribeirão Preto, 14049-900 Brazil; 2 Departments of Ophthalmology and Oncology, Wilmer Eye Institute, John Hopkins Medicine, Baltimore, USA

**Keywords:** Antiangiogenic, Autofluorescence, Choroidal nevus, Polypoidal choroidal vasculopathy, Retinal pigmental epithelial detachment

## Abstract

**Background:**

To report an association between choroidal nevus and polypoidal choroidal vasculopathy (PCV) in three patients.

**Case presentation:**

We have encountered 3 isolated patients in our center presenting with subretinal exudation and a choroidal nevus that were thoroughly evaluated by slit lamp biomicroscopy, fundus photos, Fluorescein angiography (FA), indocyanine green angiography (ICG), B-scan ultrasound, and optical coherence tomography (SD-OCT—Heidelberg). The classic features of choroidal neovascularization seen on PVC were present in all 3 patients, all of whom had a substantial response to intravitreous antiangiogenic agent. OCT, Fluorescein and ICG Angiography, and Fundus autofluorescence (FAF) revealed similar findings in all cases.

**Discussion and conclusions:**

We have identified a clinical pattern of PCV and choroidal nevus that can be diagnosed early using fluorescein angiography, ICG and OCT.

## Background

Choroidal nevus is the most common benign ocular tumor of adults [1]. Clinically, it appears as a golden-tan (if amelanotic) to dark brown flat lesion in the choroid commonly associated with changes in retinal pigment epithelium (RPE), drusen, and occasionally subretinal fluid [2]. Occasionally, choroidal nevi may be associated with the development of secondary neovascularization [3]. It is possible that the presence of a choroidal nevus may also lead to degenerative and inflammatory changes triggering the development of neovascularization.

Polypoidal choroidal vasculopathy (PCV) is a variant of choroidal neovascularization (CNV) characterized by serosanguinolent detachment of the RPE associated with a branching network of large vessels with aneurysmal dilatations [4]. PCV may occur in the macula, adjacent to the disc or in the fundus periphery. The findings suggestive of PCV observed by fundoscopy consists of red–orange lesions associated with peripapillary serosanguinolent extravasation involving the macula [5]. Injections of anti-vascular endothelial growth factor (Anti-VEGF) are used to treat eyes with PCV in hopes to maintain or improve visual acuity [6].

Optical coherence tomography (OCT), fundus autofluorescence (FAF), and ocular ultrasound are tools used to examine the morphological characteristics of PCV and choroidal nevus and making it helpful in the differentiation a benign and a malignant tumor [7].

The association between a choroidal nevus and PCV is rare [8] and some believe this is a random coincidence. We present here what we believe to be the first series of cases demonstrating the correlation between PCV and nevus in a series of patients by showing the OCT, FAF, and ICG findings involved in the development of CNV associated with a choroidal nevus and their response after treatment.

## Case presentation

### Case 1

**Fig. 1:**
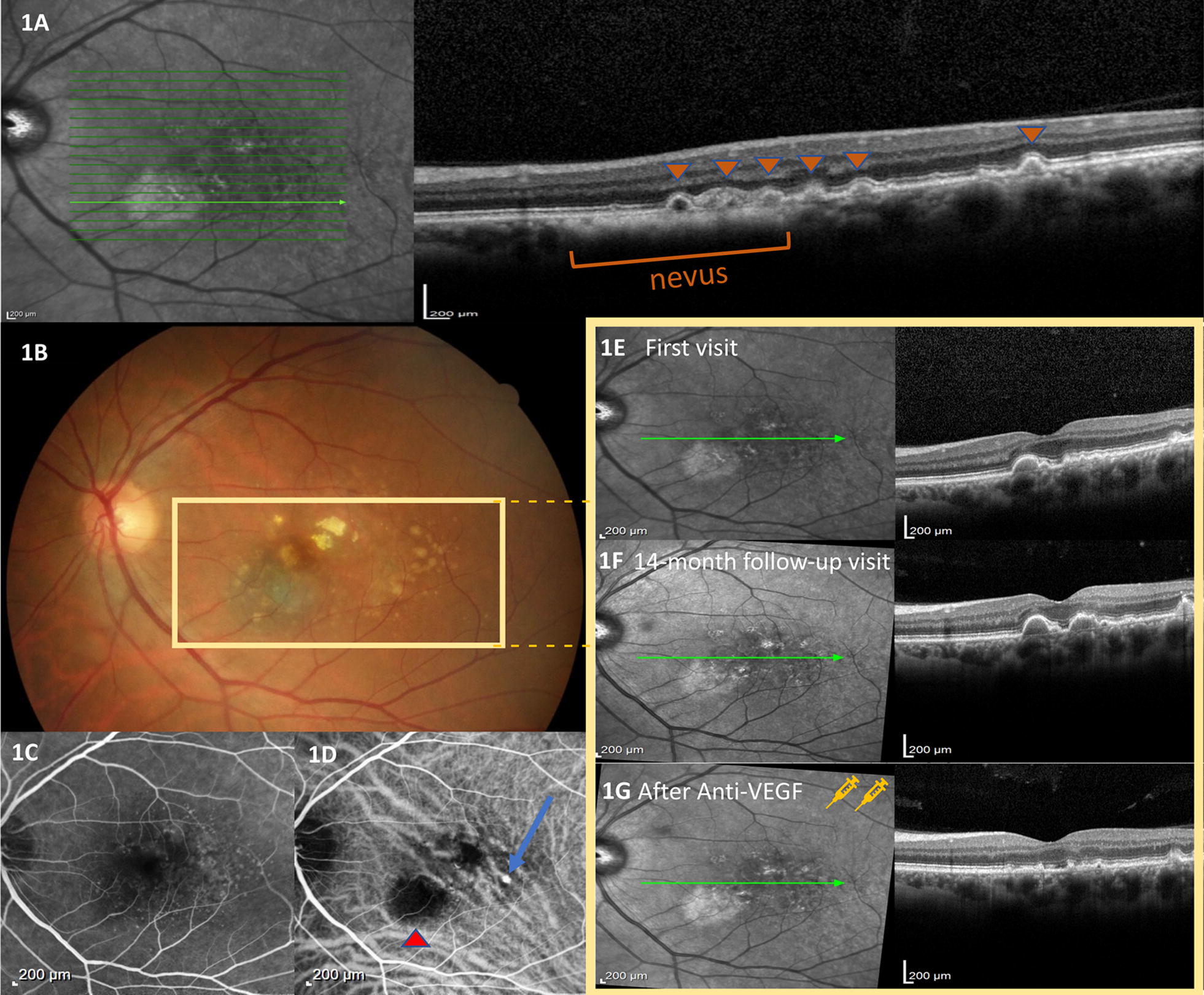
**a** OCT image demonstrating hiperrrfective changes on the choroid secondary to the nevus and also several drusen (arrowheads) over and temporal do the lesion. **b** Color fundus photograph showing the presence of nevus. **c** Fluorescein angiography and **d** indocyanine green reveals areas of hyperfluorescence (blue arrow) corresponding to polypoidal vasculopathy and an area of hypofluorescence (red arrowhead) corresponding to the choroidal nevus. The yellow rectangle shows foveal OCT images demonstrating. **e** the presence of retinal pigmental epithelium detachment in the first visit **f** its worsening after 14 months of follow-up and **g** its remodeling after 1 month of treatment with 2 monthly-bevacizumab injections

A 58-year-old woman was evaluated at the ocular oncology clinic with a complaint of floaters. Her best-corrected visual acuity (BCVA) was 20/20 (LogMAR 0) in both eyes. Retinal evaluation of the left eye revealed a flat melanocytic choroidal lesion temporal from the fovea consistent with a choroidal nevus with surface. On OCT these confluent drusen appeared as sub-RPE hyperreflective deposits. Management options included observation with self-examination using an Amsler grid and bi-annual evaluation. The patient was stable for 14 months after the initial visit when she developed metamorphosia OS causing her vision to decrease to 20/32 (LogMAR 0.2). At that time, OCT revealed multiple pigment epithelial detachments (PEDs) temporal to the nevus and ICG angiography revealed saccular vascular dilatations associated with a hot spot temporal to the fovea, compatible with PCV. The patient was then treated with monthly intravitreal injections of bevacizumab. OCT showed regression of PCV and PED after two treatments, visual acuity improved to 20/20 (LogMAR 0) OS. (Figure 1).

### Case 2

**Fig. 2:**
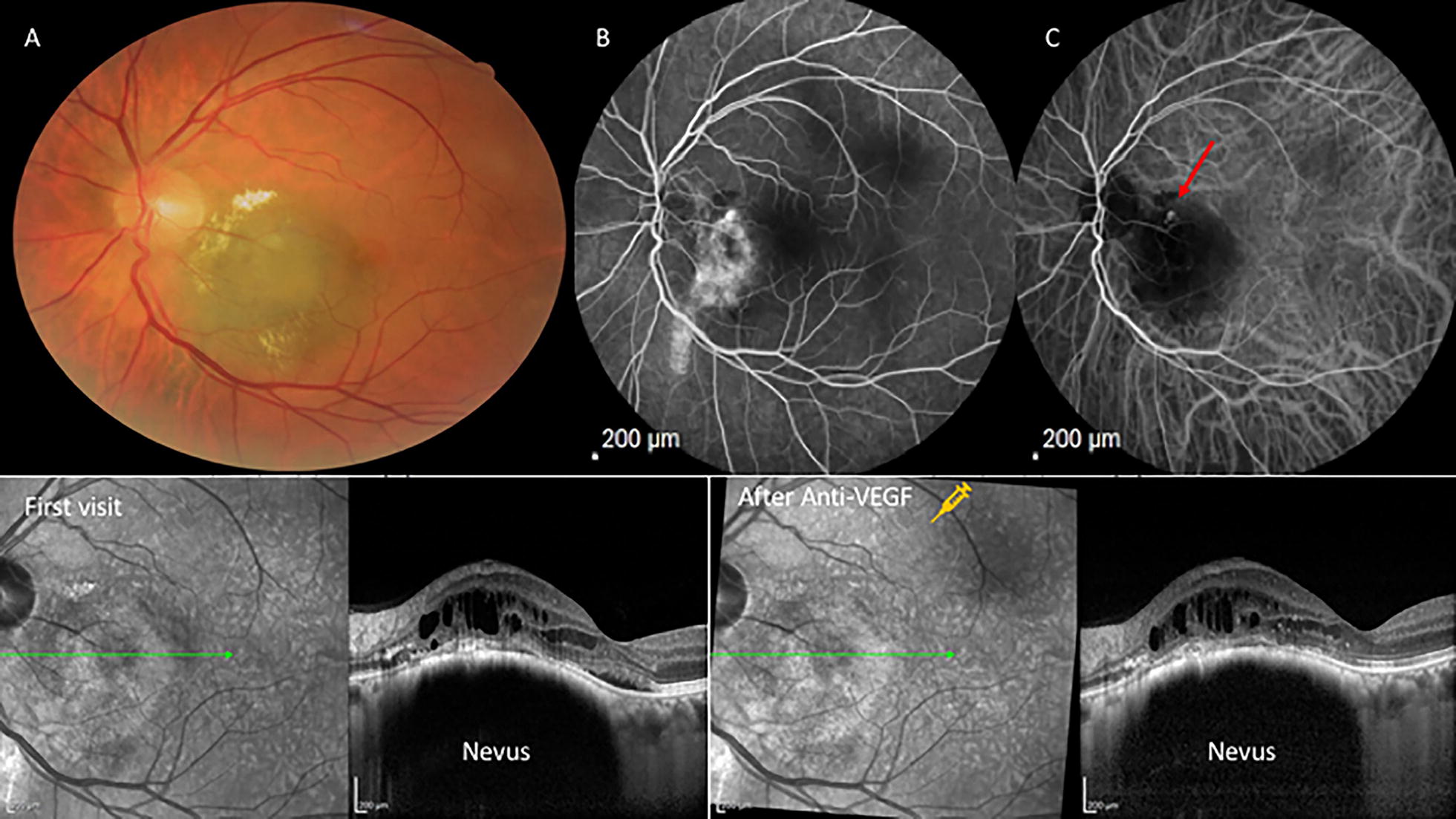
**a** Color fundus photo shows an elevated hypechromic lesion consistent with a choroidal nevus. **b** Fluorescein angiography shows an area of peripapillary hyperfluorescence. **c** Indocyanine green angiography shows a hot spot (red arrow). Bottom: **d** OCT images demonstrating the presence of subretinal fluid in the first visit and **e** shows the response after 1 month of one ranibizumab intravitreal injection

A 60-year old man complained of progressive worsening of this vision OS for the past 5 years. Ophthalmic examination revealed BCVA OD to be 20/20 (LogMAR 0) and LogMAR 0,3(20/40) OS. Fundus examination showed an elevated melanocytic choroidal lesion OS associated with hard exudates and surrounding sub-retinal fluid. OCT-EDI (Enhanced Deep Image) revealed the presence of edema overlying the hyperreflective choroidal lesion. Fluorescein angiography revealed delayed leakage corresponding to the region of the lesion, and ICG showed the presence of polypoidal vasculopathy during early/middle phase. Ranibizumab was then injected intravitreous twice with a 1-month interval between them. The patient returned for a follow-up visit showing vision improvement to LogMAR 0,1(20/25) and resolution of subretinal fluid (Fig. 2).

### Case 3

**Fig. 3:**
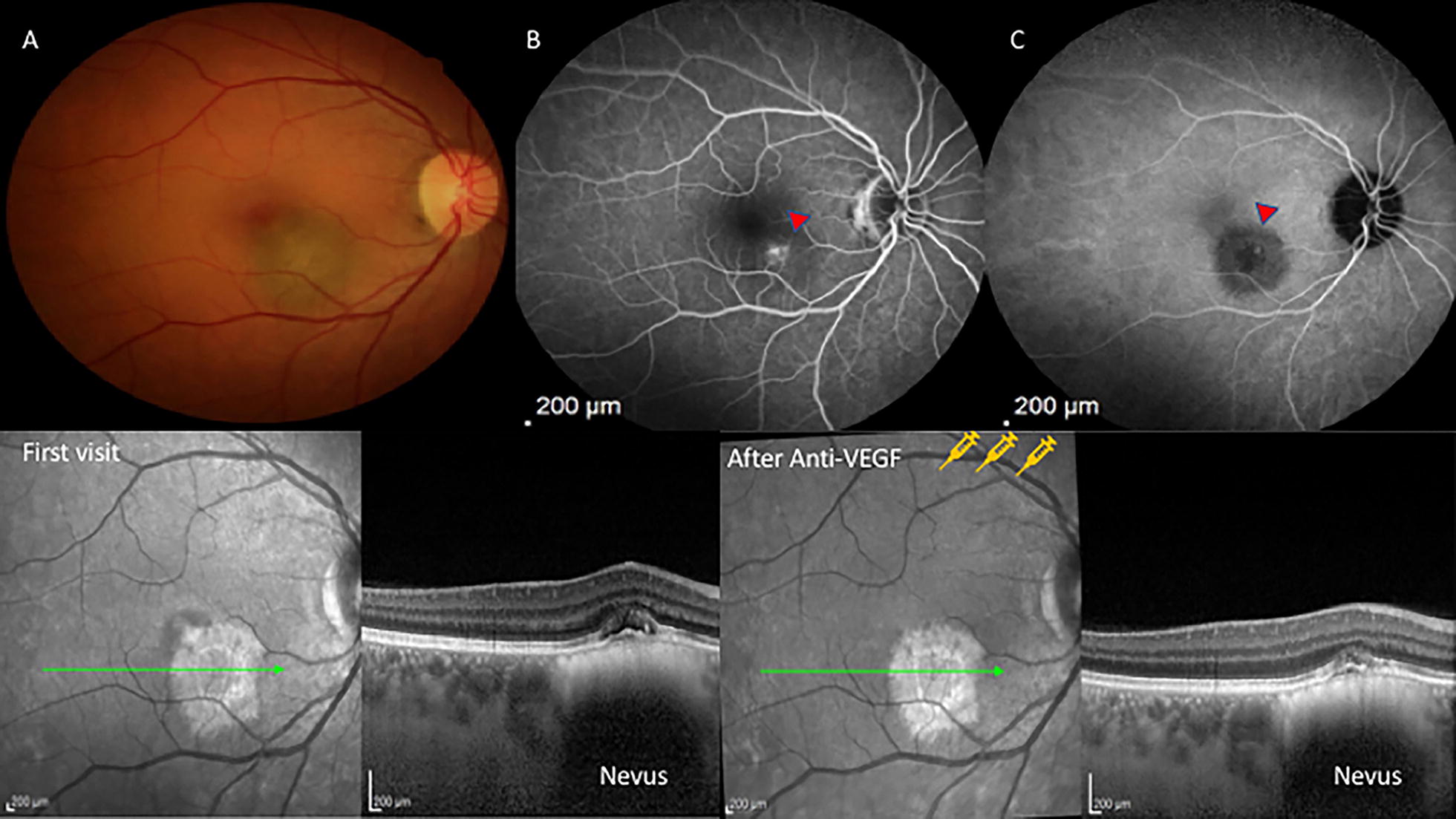
**a** Color fundus photo shows an elevated melanocytic choroidal lesion consistent with a nevus. **b** Fluorescein angiography shows an area of hyperfluorescence on the surface of the nevus. **c** Similarly, indocyanine green angiography shows a hot spot (red arrowhead) on the nevus’ surface corresponding (PCV). Bottom: **d** OCT images demonstrating the presence of subretinal fluid and loss of integrity of internal retinal layers in the first visit and **e** shows the treatment response after 1 month of 3 monthly-bevacizumab intravitreal injections

A 53-year-old woman complained of decreased visual acuity in her right eye of 3 weeks duration. Examination revealed BCVA to be LogMAR 0,1(20/25) OD and LogMAR 0(20/20) OS Fundus exam revealed a melanocytic choroidal tumor measuring 2.5 mm on basal diameter associated with perilesional edema. OCT confirmed the presence of subretinal fluid. Fluorescein angiography revealed a hyperfluorescent lesion with leakage in the late phases. ICG revealed a small round hyperfluorescent spots consistent with PCV overlying a choroidal nevus. The patient was managed by monthly intravitreal bevacizumab for three months. The patient showed substantial response with vision improving to LogMAR 0(20/20) and resolution of subretinal fluid (Fig. 3).

## Discussion and conclusions

The association between a choroidal nevus and PCV is a rare but important condition [9] since it may cause substantial vision impairment as illustrated in this case series.

Choroidal nevi are known to cause remodeling of photoreceptors early on, although usually not causing vision loss. [10] Studies found in the peer-reviewed literature have shown central vision loss and visual field defects related to the presence of a choroidal nevus in 10 to 38% of the cases [11]. Similarly, subfoveolar location of the nevus, as shown in this series, is a risk factor for central visual acuity loss over time, even without PCV [12]. It makes sense that, in most of these cases, vision loss related to foveal location is related to the presence of chronic subfoveal fluid and photoreceptor degeneration.

Our group has shown that a choroidal nevus can lead to chronic changes in the overlying retina [10], and these changes may lead to reduced sensitivity in microperimetry (unpublished data). It seems that a choroidal nevus can mechanically damage the choriocapillaris and lead to chronic changes such as drusen and choroidal neovascularization. In fact, there has been speculation that patients with choroidal nevus and PCV might have had chronic/secondary degenerative or inflammatory changes of the RPE. Further, such changes might result in the growth of Type 1 neovascular membranes and eventually in the development of PCV adjacent to the melanocytic lesion, as we have observed in this case series. In addition, another possible hypothesis would be the anecdotal association of PCV and choroidal nevus in the context of paquichoroid(Anzidei) [13].

The clinical course of our patients confirmed that the associated neovascularization caused vision loss similar to the observation of Asao et al. [3]. Newer technologies such as OCT, FAF and ICG angiography have allowed earlier detection of choroidal neovascularization associated with choroidal nevus, as demonstrated in our series and reported by other in the peer reviewed literature [3, 7].

Photodynamic therapy was the first proposed treatment for neovascular membrane associated with choroidal nevus that provided long-term visual preservation as originally reported by Querques et al. [14]. However, since the development of anti-VEGF agents, more specifically aflibercept, patients with PCV were not only able to experience visual improvement but also demonstrated regression of neovascularizations for long periods of time, as observed in the present case series and reported in cases of PCV [6].

The present study has some limitations such as its small number of patients and its retrospective/descriptive nature. However, the rarity of this association poses a challenge to recruit a larger number of participants. Our results show that intravitreal anti-VEGF can be effective to treat PCV associated with choroidal nevus. The importance of close follow-up with careful monitoring of these eyes relates to timely indication of intravitreous anti-VEGF agents. Additional long-term prospective studies are needed to determine potential causes for this association, clinical course and visual acuity of patients with choroidal nevus and PCV.

In conclusion, our experience has shown that FAF, OCT, FA, and ICG are useful tools to identify and characterize choroidal nevus with secondary CNV and to monitor the effectiveness and response to intravitreous anti-VEGF agents in reducing exudation and improving visual acuity.

## Data Availability

The datasets used and/or analyzed during the current study are available from the corresponding author on reasonable request.
